# Path to European quantum unicorns

**DOI:** 10.1140/epjqt/s40507-021-00095-x

**Published:** 2021-02-25

**Authors:** Markku Räsänen, Henrikki Mäkynen, Mikko Möttönen, Jan Goetz

**Affiliations:** 1 IQM, Keilaranta 19, 02150 Espoo, Finland; 2 Present Address: QCD Labs, QTF Centre of Excellence, Aalto University, 00076 Aalto, Finland; 3 VTT Technical Research Centre of Finland Ltd., POB 1000, 02044 VTT, Finland

**Keywords:** Quantum computing, EU, Unicorn, Industrial policy

## Abstract

Quantum computing holds the potential to deliver great economic prosperity to the European Union (EU). However, the creation of successful business in the field is challenging owing to the required extensive investments into postdoctoral-level workforce and sophisticated infrastructure without an existing market that can financially support these operations.

This commentary paper reviews the recent efforts taken in the EU to foster the quantum-computing ecosystem together with its current status. Importantly, we propose concrete actions for the EU to take to enable future growth of this field towards the desired goals. In particular, we suggest ways to enable the creation of EU-based quantum-computing unicorns which may act as key crystallization points of quantum technology and its commercialization. These unicorns may provide stability to the otherwise scattered ecosystem, thus pushing forward global policies enabling the global spread of EU innovations and technologies.

The unicorns may act as a conduit, through which the EU-based quantum ecosystem can stand out from similar ecosystems based in Asia and the United States. Such strong companies are required because of the level of investment currently required in the marketplace. This paper suggests methodologies and best practices that can enhance the probability of the creation of the unicorns.

Furthermore, we explore future scenarios, in which the unicorns can operate from the EU and to support the EU quantum ecosystem. This exploration is conducted focusing on the steps to be taken and on the impact the companies may have in our opinion.

## Introduction

Quantum technologies and their derivatives such as quantum computing are a field with great future promise. Europe, and more importantly the countries within the EU have globally leading academic teams in quantum-computing science. However, the desired future large-scale commercialization of quantum-computing technologies in Europe calls for certain shifts in industrial, academic, and innovation policies. Most importantly, top quantum technologies must now make the transition out of academic research groups into industry to ensure that Europe can reap the rewards for the decades of systematic funding of basic research in this field [1].

To commercialize quantum computing in Europe, there is a need for so-called unicorn growth companies [2]. These significant scale-ups are companies that are on their way growing from startups into major international companies. They are important because of their sufficient size to stand out and succeed on a global scale. It is imperative that we in the EU aim for the creation of such companies since only with such seeds of success leading the way, the EU can stay globally prosperous and ensure access to emerging technologies.

We explore the topic of quantum unicorn companies in this commentary arguing that their successful creation calls for certain key changes and additions in the existing funding instruments towards the commercialization of quantum-computing technologies.

## Commercialization of quantum-computing technologies

Quantum-computing technologies address various computational problems unsolvable by current computing hardware such as the simulations of complex phenomena in chemistry, finance, and other fields [3]. The quantum-computing market size has been estimated to grow to above six billion euros in 2027 globally [4]. However, current quantum-computing systems cannot yet solve viable business problems.

Therefore, most current customers for quantum-computing hardware consist of government-owned research institutions. Their motivation is, for example, to train next-generation quantum engineers, to be frontrunners in using the technology, and to explore the future potential of quantum computers in the fields of chemistry, finance, and optimization methods [5]. This research-based market is complemented by consultancy services for decision makers to make their organizations quantum ready and by proof-of-concept tests of cloud computing services. Large-scale commercialization in the field is expected once quantum computers reach the first quantum advantage, i.e., they outperform in some way any classical computer in a computational problem of practical value.

With the arrival of quantum advantage, the market for quantum computing is likely to expand dramatically, quickly multiplying in size. In addition, the arrival of quantum advantage is likely to create new markets. Since the potential of quantum computing can unlock problems previously unsolvable and hence create its own application areas, it is hard to estimate its future potential market size. However, the market may become on par or even larger than the current high-performance computing market.

Currently, the quantum-computing technology and intellectual property (IP) developed in academia are being utilized in multiple startup companies as the field has entered a phase where the innovations from academia move to the industry. It is estimated that Europe alone has more than 69 quantum-computing startups founded since the year 2010 [6]. The total number of European quantum startups is likely to be much larger, but the exact amount is hard to verify since it also depends on the definition of a quantum-computing startup. Nevertheless, this number is high on a global scale. For example, it has been estimated that the number of quantum-computing startups in the United States is at 53 currently [7]. The difference can be potentially explained by considering the quality and quantity of the European academic research working on quantum technology. According to a 2017 paper, 2455 authors of quantum physics papers were from the EU, 1913 from China, and 1564 from North America [8]. Although this is a reasonable indicator, as we will show below, the quality of the companies and the amount of funding as well as governmental support available is more important for finding out the quantum-computing companies that may become global leaders in their domain.

Quantum computing is a particularly capital-intensive field. Depending on the technology platform, the equipment used to operate the quantum processor may cost more than million euros even for initial research machines. This combined with the personnel costs, the costs of facilities to manufacture the components such as quantum processing units, that may form a key part of the products of the startup company, may well lead to a situation where a startup company needs tens of millions of euros to efficiently start up, and much more for continued operation.

This type of cost base potentially leads to a situation where the valuations of quantum startups need to increase in a rapid manner to keep up with the funding needs of the business. As such, the key issue we are addressing and arguing in this commentary is that the EU needs to foster the creation of quantum-computing unicorns in order to be in an advantageous position to benefit from the expansion of the quantum industry. Preferably many of such unicorns will be present in the market at the time of its initial rapid expansion.

### Commercialization of quantum-computing technologies in Europe and the related challenges

As discussed above, quantum computing technology, like many other deeply technical fields, requires large amounts of capital and proper infrastructure to be successfully commercialized. We estimate that the commercialization of quantum-computing technology to a sufficiently strong level globally requires a total EU-wide investment of roughly 15 billion EUR between the years 2020 and 2030. Our estimation is based on the already published quantum related programs by other countries as well as the current state and the monetary requirements of the field [9].

One of the key areas of improvement is the speed, at which quantum startups move from academia to businesses. This is a self-amplifying process: An overall increase in this speed is prone to create more such companies, leading to increased funding flowing into quantum-computing technology. Another argument is the access to organizations, whether government or privately owned, that are willing to co-operate with these early-stage companies. Such willingness can be characterized as very early adoption of new technologies in the form of pilots and other such projects versus waiting for more mature solutions.

Although the research community in the EU is currently on par with the communities in the United States and China, the commercialization indicators are already lagging. One of these indicators is the number of filed patents.

**Figure 1:**
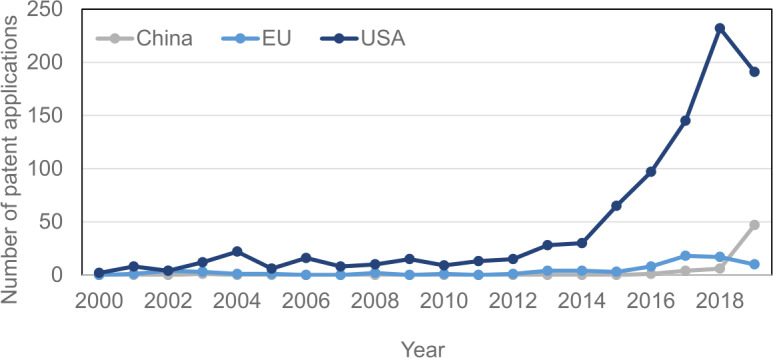
Number of annually filed patent applications in the patent class G06N-010/00 (“Quantum computers, i.e., computer systems based on quantum-mechanical phenomena”) as a function of time by country or area as indicated. The data are obtained from Reference [10]

Currently, the United States is the clear leader in the number of filed quantum-computer patents followed by China (see Fig. 1). Note that the Chinese effort is significantly encouraged by the government subsidies for patenting [11]. Europe’s ranking leaves room to improve, for example, with more well-funded quantum startups or stronger incentives to patent the technology. There are likely correlations between the patent-filing figures and investments into quantum computing and quantum technologies in general. Nevertheless, the EU-based companies or institutions are not patenting on the level they should be considering the amount of leading academic research going on in this field in the EU.

We consider that part of the above-mentioned lack of patenting is explained by a shortage of large enough quantum-computing companies applying for patents in the EU. Large companies can spend major resources in patenting and generate large patent portfolios, which is challenging for small and medium-sized enterprises (SMEs) or startup companies. Therefore, we suggest the following measures to promote Europe in terms of protecting IP:

1. Create awareness among scientists in universities and research organizations that filing patents is essential for technology building and that filing patents is compatible with publishing the results also in scientific journals.
2. Create innovation hubs in universities and research organizations to educate their scientists about the benefits of patentable results to create a pull effect for patents.
3. Establish IP pools where patents of different groups can be submitted to and where companies and startups have access to.
4. Simplify the spin-out process for IP based on lump-sum payments to support the creation of startups owning a reasonable amount of IP when starting the operations.
5. Subsidize the creation of patents for startups to make sure that their liquidity is not the limiting factor to file patents.

### What is needed to create business around quantum technologies?

Funding is necessary, but not the solution to all problems. Quantum-computing companies need significant funds to be able to spin out from academia. Although the EU has substantially added funding for technology startups, most funding is still geared towards investments that are considered by the private investors to be simple and of not of very high risk, such as investments in standard software companies.

**Figure 2:**
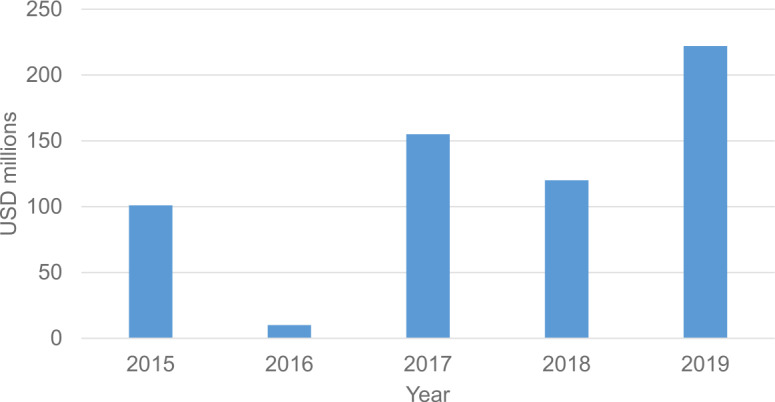
Capital invested in quantum-technology companies globally each year. The data are obtained from Reference [12]

The global trend towards more capital investments in quantum-technology companies, not just those in quantum computing, is promising as shown in the Fig. 2.

**Figure 3:**
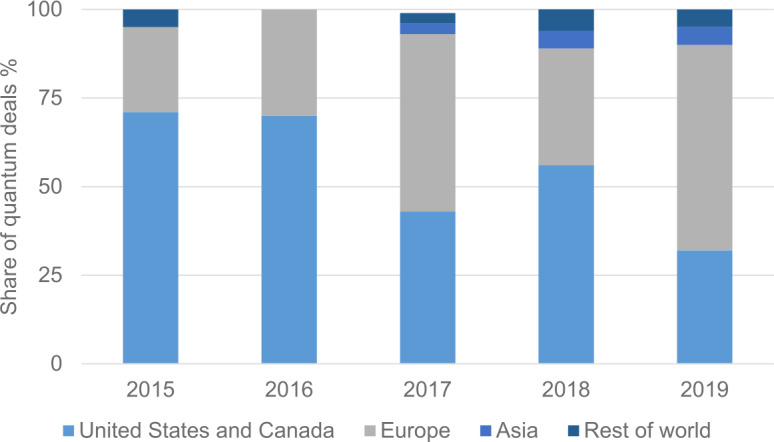
Relative regional distribution of the number of investments in quantum-technology companies realized each year. The data are obtained from Reference [13]. Note that the data for China are embedded in the figures for Asia and the figures are not available separately

Figure 3 shows clearly that the number of quantum-related investment rounds in Europe has grown significantly, covering more than half of them in the world in 2019. However, Europe needs to ensure that the positive trend continues. In addition, having only small startups does not create a leading position for Europe, and hence the main focal point of this paper is how to create unicorns and scale-ups out of the present situation. Unfortunately, in the number of all types of scale-ups, Europe is lagging strongly behind the US [14].

Although drawing the distinction between private and public funds is important, we stress that as one of the most significant economic actors globally, the EU has enough private and public capital to fund significant projects such as the commercialization of quantum computing by startup companies [15]. The combination of private capital and public funding is essential for any venture in quantum computing to succeed. Although public funding in the EU has produced significant scientific results in the past, we consider that the commercialization of these scientific results has been less active than that, e.g., in the United States [16]. With certain adjustments, the speed of commercialization and the number and size of companies can be increased.

In addition, quantum computing companies face a general challenge: quantum advantage is still to be achieved in the future, but sales should be realized now. Since the current-generation quantum computers are best suitable for research and education, governmental organizations are the natural first customers. They have a major interest in research and education, typically enforced by legislation. Furthermore, governments typically have the role of taking this kind of long-term financial risks since it is beneficial from an economic point of view. Creation of new industries brings in the long-term major returns to the governments in the form of taxes and efficiency. The private industries in Europe active in the sectors where quantum computing has the greatest potential, need to also consider investing in such machines already now, for example, to educate their workforce and to polish their solutions to be ready for immediate utilization in the beginning of the quantum-advantage era. Having this talent in combination with achieving quantum advantage is an invaluable asset for such businesses.

### What are the limitations in Europe?

**Figure 4:**
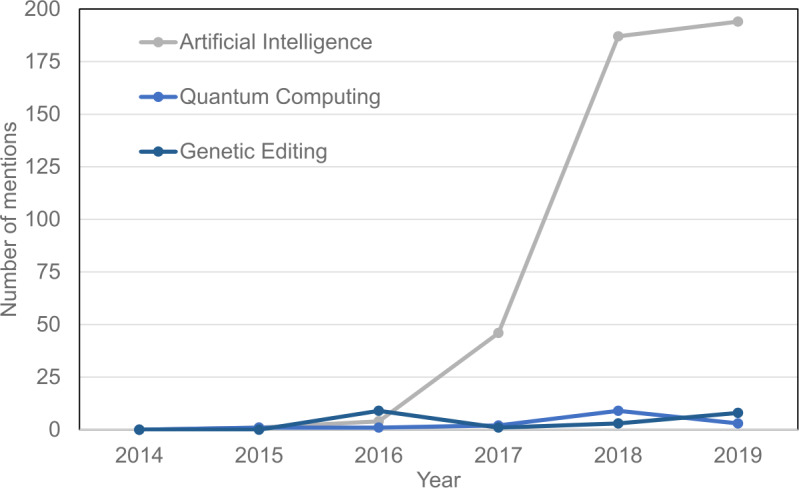
Number of mentions of Artificial Intelligence (grey color), Quantum Computing (light blue), and Genetic Editing (dark blue) in European Parliament activities and press releases each year. The data are obtained from Reference [17]

**Figure 5:**
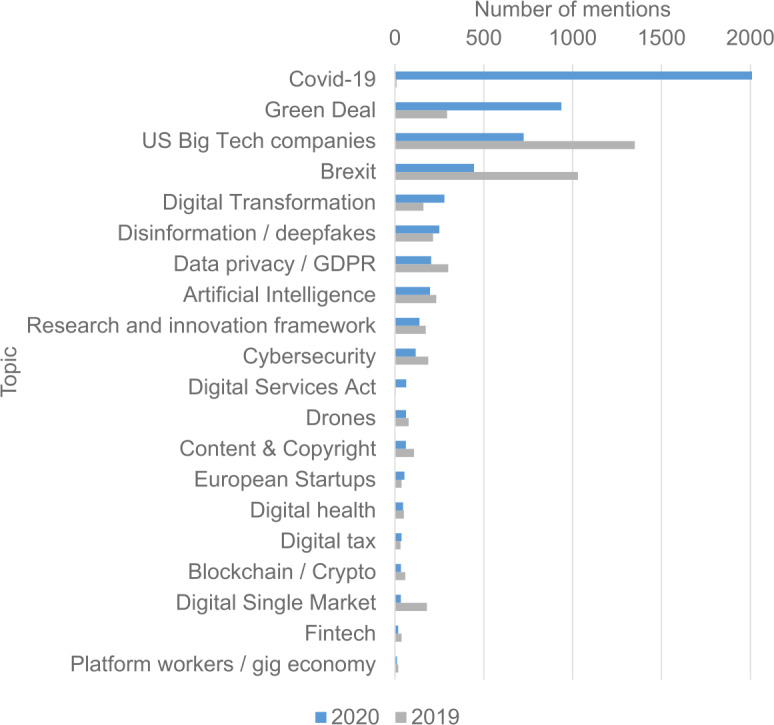
Top 20 key topics in European Parliament by number of mentions in activities and press releases for 2019 (grey bars) and 2020 (blue bars). The data are obtained from Reference [18]. Note that the data for Covid-19 exceeds 5000 mentions

The European Commission and the European Parliament play a significant role in setting the high-level agenda that will be adopted into the local agendas of the member states. Unfortunately, as shown in Figs. 4 and 5 below, quantum computing is not yet among the popular topics of discussion in the European Parliament. Figure 4 shows the position of quantum computing as a topic versus other emerging technology topics and Fig. 5 shows the most frequent discussion topics regarding the technology industry in the European Parliament.

Thus, we stress that the European Parliament should more closely examine the recent developments in quantum computing and consider questions such as technological sovereignty. A future implementation of a strong initiative of the European Parliament in raising awareness in quantum computing and technology has the potential to create positive impact towards the success of the European quantum-computing companies. Such discussion is prone to affect also the national political discussion.

**Figure 6:**
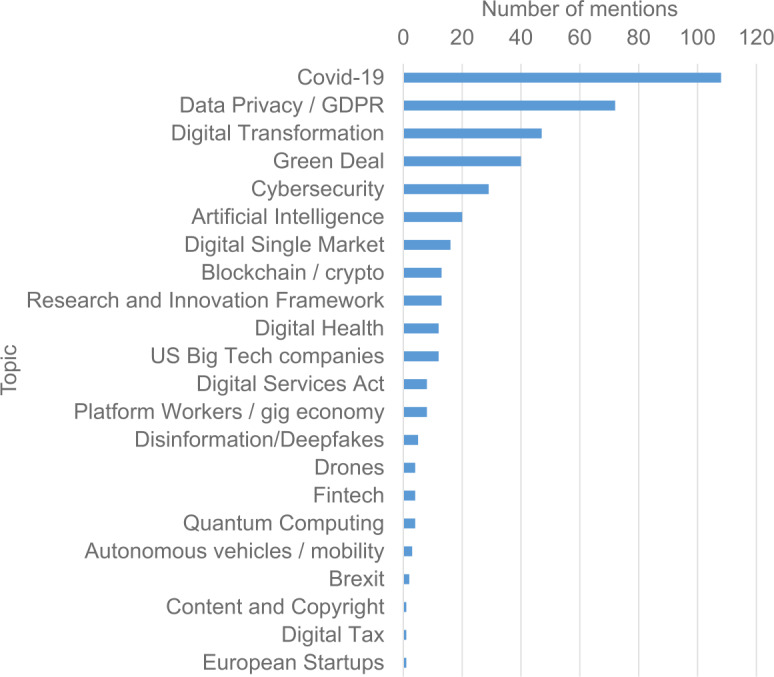
Number of mentions of key technology-related topics in European Parliament legislative documents by topic in 2020 in a descending order. The data are obtained from Reference [19]

Although some improvement has taken place in terms of bringing quantum computing to the legislative agenda, as shown in Fig. 6, there is still room to improve. The related limitations in the EU lie in the lack of cohesion for the public and private actors to bring capital-intensive solutions to the market. There are examples such as Airbus, where European countries have formed a major global company to meaningfully operate in an extremely capital-intensive field, but we think that more such companies can emerge when the public and private sides work together.

One of the pressing challenges and limitations to the growth of significant global companies out of the EU in capital-intensive fields is the non-ideal machinery of the European Union to act as the first-instance buyer. The United States has governmental initiatives such as DARPA which act as the first buyer for solutions emerging from basic research [20]. Such buyers are important for the development of industries that are both risky and require significant capital. There are some indications that the European Commission will purchase European quantum computers as per some of the draft plans in the European High Performance Computing Joint Undertaking Programme, but the details of these plans are not yet public. Only a proposal regulation is out at the time of the writing of this paper [21]. This is a good direction for future developments, but discussions with the European industry and stakeholders needs to be enhanced to ensure that the investments have the desired impact.

Furthermore, although the research funding is currently on a healthy basis in the EU, there is discussion on potentially cutting the EU research budget such as that of the Horizon program. However, such cuts seem not to have materialized to their full extent, for example, due to the COVID-19 crisis [22]. Cutting is the opposite of what the EU needs at this stage. Only continued commitment and investment into science can produce the required level of basic research which can then be commercialized by companies within the EU.

Naturally, the EU is comprised of various member states and there are differences between the approaches taken and results achieved between the states. However, for any globally relevant moonshot project like quantum computing, the involvement of the EU is required. An analogy of this is the establishment of the European Space Agency as opposed to individual European nations having their own space programs. There are several other similarities between the space sector and quantum technologies. One notable similarity is that both fields may bring unforeseen applications such as solar cells emerging out of space-related research. In this context, funding of quantum technology may generate many-fold returns for the investment.

**Figure 7:**
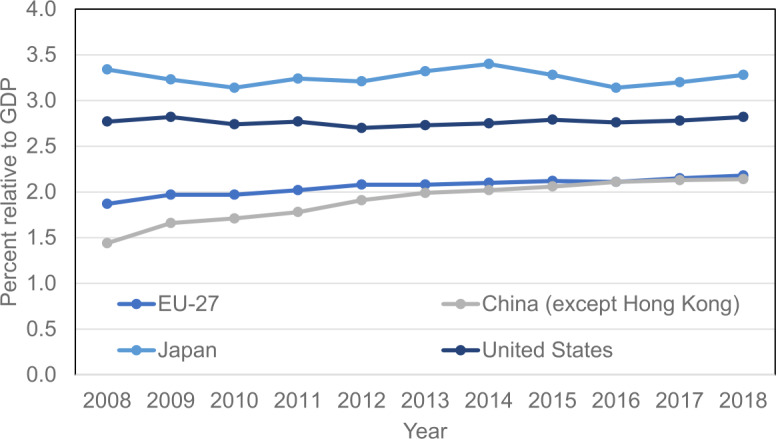
Gross domestic expenditure on research and development for 2008–2018 in each region as indicated. The data are obtained from Reference [23]

One of the most alarming indicators where the EU is lagging is the amount of research and development (R&D) expenditure relative to the gross domestic product (GDP), as shown in Fig. 7. Furthermore, it has been argued that a leading company or the leading few companies may capture most of the relevant market [24]. This leads to the ability of the largest companies to spend significant resources in R&D. On the country level, this phenomenon has been observed in Finland, where after Nokia reduced its R&D expenditure, it significantly affected the national expenditure level [25]. Thus, it may be that if the EU is not active in the development of quantum computing and other future technologies, a similar development may happen on the European level and affect the European competitiveness potentially for decades.

**Figure 8:**
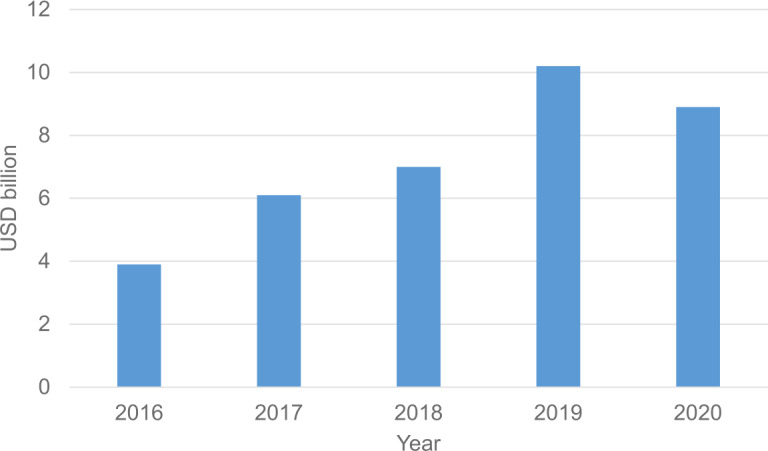
Capital invested in European deep-technology companies in each year. The data for 2020 are normalized for the full year based on data until September 2020. The data are obtained from Reference [26]

The EU can also play a significant role in encouraging private capital investment into deep-technology companies. Of course, part of this work will be carried out by the individual member states, but as an EU-level action is of great importance since as Fig. 8 shows that there is a potential slump in the amount of funding for such companies in Europe. However, the data are currently inconclusive as to whether the growth of funding will again continue in 2021.

Much of the potential economic capture will not be realized in the EU without significant investment into new quantum-computing startups and bringing these startups to the level where they can stand out as global leaders in their field. In essence, Europe is paying for the ground-breaking science, but may not be able to reap its full benefits later. Only the major unicorn-like startups can grow to the scale required for the massive ecosystem defining R&D investments. Only large enough companies can make the investments required to boost the whole EU quantum-computing ecosystem.

## Current solutions and related commentary

When allocating large amounts of capital to support deep-technology developments as in the current situation, it is of utmost importance to have a strong resource allocation plan for the investments. Although the EU is still behind, e.g., the United States in funding moonshot projects such as quantum computing, with a proper plan such gaps can be removed. If the EU continues to view quantum computing as an area of strategic importance, as it has done thus far, they should be willing to further invest into the commercialization of this technology, greatly exceeding the current levels such as those of the Quantum Flagship [27].

It is known that the EU has been left outside major technological value creation ecosystems such as various internet platforms due to the lack of EU-based companies offering a challenge to, e.g., United States-based and Chinese internet companies. A relevant question in this context is to what extend in the long term the EU economy can handle the scenario where new technologies are commercialized elsewhere. Below, we present some views on solutions to this dilemma such as how to enable the creation of quantum-computing unicorns in Europe.

### Preparing the ground: research flagships

As mentioned above, the concentrated effort in quantum computing in the EU is referred to as the Quantum Flagship. The importance of such programs manifests in fostering the cross-border co-operation between research teams, both academic and corporate, in different countries, companies, and universities. However, the purpose of such flagships should not be to compete or even try to be an alternative to commercial solutions, but to enable increased technology transfer from academia to industry. We stress that forced commercialization is not the way in which Europe will achieve the goal of creating world-leading quantum unicorn companies. Instead, competitive business models should be supported that allow startups to scale and become significant among the technology giants in the US and Asia.

The research flagships such as the Quantum Flagship play a key role in displaying to the community what is important to focus on in the future. Still, the EU should not create a situation where these initiatives are competing against EU-based companies. Flagship projects should develop technology that can be commercialized in a subsequent step by startups and industry. Therefore, we propose that the Quantum Flagship and similar pan-European research activities focus on ways to enhance the capabilities of EU-based quantum companies by conducting application-driven research. The results of such research should be protected by intellectual property licensing with agreements that are attractive to the commercial community. A model where government-funded research organizations build their own patent portfolios to block European companies from using the related technology should be discouraged.

### Driving innovation: quantum hubs

To succeed, massive moonshot projects require concentrated funding. The increased complexity of technologies coupled with the increased global competition requires that EU funding is focused on fields that are critical for future European competitiveness such as quantum computing.

We propose the EU to fund spearhead research centers of excellence in key locations in the EU to increase the quality and quantity of results. For quantum computing, such centers, or quantum hubs, can be in critical locations where the next generation of quantum scientists are trained, and they can also act as technology hubs fostering further commercial innovation.

By driving innovation and supporting spinouts, such centers can serve the creation and continued growth of leading quantum unicorn companies by acting as sources of talent, funding, and co-operation projects. The centers can also act as a purchaser of equipment from EU-based startups and scaleups, supporting them to become quantum unicorns. The centers do not compete with the local or other EU-universities, instead they should work together with and are coordinated by universities, startups, and industry.

It is also very important that these centers concentrate on the commercialization of the results of the local and the European academia. Just supporting academia will not bring commercial traction. Instead, the technology developed in the academic groups needs to be commercialized in a manner which produces further economic prosperity such as jobs or exported goods.

### Supporting commercialization: industrial subsidies

We have identified the offering of industrial subsidies to companies in field of quantum computing as a solution to increase the overall commercialization of quantum-computing technologies in the EU. Such subsidies can be offered based on the EU and local law by the member states in the form of research grants, tax incentives, or by other allowed means. We think that these subsidies will encourage large companies to acquire products and technologies from EU-based quantum-computing companies. Such subsidies will also encourage EU companies to adopt technologies quicker. These large companies can play a major role in the commercialization of quantum computing technologies as they are very sophisticated customers. In order to build solutions that these companies can use, the EU-based startups will need to perform at a level that can boost further success.

Although subsidies are a controversial topic, they can be extremely helpful in supporting specific investment-heavy industries such as quantum computing. We stress that the controversial nature of subsidies implies that they should be as focused as possible. For example, subsidies may be offered for the cooperation between EU-based quantum-computing companies and EU-based pharmaceutical companies for certain use cases such as the discovery of critical pharmaceuticals or therapies.

Naturally, the quantum computing field cannot be driven by subsidies alone. We want to stress the importance of proper business models for quantum-computing companies since having such a business model with persistent growth of revenue is one of the best ways of gaining the unicorn status. Also, the business model itself or the technology driving the business needs to be superior compared with the existing and emerging companies.

## New solutions: next-generation public private partnership

The strong commitment to fundamental scientific research and engineering in Europe has proven successful many times in the past. The cooperation between the research community and commercial industries has yielded supersonic commercial airplanes, bullet trains, and the massive European-lead international collaboration to build the largest and highest-energy particle collider in the world, the Large Hadron Collider.

Taken that the EU decisively built, for instance, the above-mentioned significant global aerospace pioneer Airbus, similar successful cooperation between the research and commercial industries can be applied to build quantum computers as well. However, a careful comparison of the Airbus case with the current situation in quantum computing reveals a striking difference, namely, that the commercialization in quantum computing is not as advanced in terms of the stage of commercialization as the commercialization of passenger aircraft technology was for the founding members of Airbus. Therefore, one must take the best parts of the public-private partnership model to create a critical mass of commercially successful quantum startups. This group of startups and the already established companies can lay the ground for the creation of quantum-computing unicorn companies in Europe and lead to an organization that can be considered a quantum Airbus.

Below, we outline certain areas of the recent and future public-private cooperation that we think are suitable for the creation of quantum-computing unicorns in Europe.

### Technological excellence: lighthouse projects

As stated in Sect. 3.2, the creation of world-class quantum hubs or centers of excellence is an extremely promising area of public-private partnerships. Promoting this idea further, these centers of excellence could also actively participate with the local industry in guiding them towards future use cases of quantum computing. This activity may lead to commercially useful quantum computers, which may be considered as an European lighthouse project.

Therefore, we propose that the EU sets up 3–5 such lighthouse projects in those EU cities or regions with existing strong quantum-research traditions and then increasing the number or volume as the importance of quantum technologies increases. These centers can also host infrastructure required by startups such as the quantum computers themselves as well as manufacturing and testing facilities required for prototypes.

Such lighthouse projects can be funded in a shared manner by the EU and the local country or a group of local countries. They may be tuned into the local industrial base by having continuous dialogues with the local companies on how these companies can use quantum computing and what their needs are. Naturally, we advocate for a hybrid model between academia and industry. In the best case, such a hub can add many more talented researchers and quantum business professionals to its location as well as increase the number of startups due to the concentration of both talent and resources.

### Made in Europe: access to manufacturing infrastructure

The presence of major centralized infrastructure is a critical aspect required for the creation of serious, especially unicorn-status quantum-computing companies. Typically, the building and creation of infrastructure such as cleanrooms will take too long to fit into the schedule of a fast-growing startup and such facilities may not be supported by the venture-capital investors.

We propose that the EU funds the creation of critical infrastructure required by quantum-computing startups especially in those regions with nascent ecosystems and reasonable existing infrastructure. This critical infrastructure can take the form of cleanrooms whether updates to existing ones or new ones, as well as manufacturing facilities for larger-scale manufacturing of quantum-computer parts such as quantum-processing units. The successful manufacturing also calls for testing facilities hosting many independent, but to a certain level, standardized setups.

There is a special need for cleanrooms for small-scale production of prototypes required during the development and scaling years of any quantum-computing startup towards the unicorn status. Concretely, such a cleanroom project can take a form where EU funds are used to coordinate the building of such a cleanroom building with enough space leasable exclusively for companies as well as with space and equipment in shared use. The building itself is typically the highest concern since if it does not already exist as a cleanroom, its construction may be prohibitively expensive for any single company.

The equipment in such a cleanroom is another major hurdle. Startups and non-established companies struggle to acquire credit or bank loans for the purchase of the needed equipment. One way to solve this problem is to have selected shared equipment in the cleanroom where feasible and to offer guarantees for companies for purchasing their own exclusive equipment to be housed in such a cleanroom.

### The SpaceX business model: public procurement projects

For many new complex technologies, the first purchaser will often be a state since the business may be too risky for the free market. Quantum computing is no exception here since the commercial use-cases still need to be verified. However, for startups showing commercial traction public procurements are essential since the first commercial sales are the most important in any startup journey. In the case of quantum unicorns, it is critical that during the nascence of quantum computing, the EU and the EU member states support the EU quantum-computing companies such that they can take a strong position until the wide market for quantum-computing solutions opens.

We propose that the EU encourages its own agencies and member states to purchase quantum computers from EU-based quantum-computer companies instead of only providing research grants. This business-centric approach is beneficial in encouraging the quantum-computing companies to act in a way that produces commercial results in a timely manner opposed to only carrying out research for products in the far future.

The quantum computing infrastructure built in this way enables the creation of additional quantum-computing companies as well as the overall development of the field within the EU. The infrastructure also brings more people into the field and with more people as well as with the increase in infrastructure utilization, more innovation is expected to follow in fields connected to quantum computing such as software and services. These public procurement projects will ensure that the coordination of the future of the quantum industry rests with the quantum industry itself and that commercialization is the driver of the agenda.

### Build ecosystems around startups

**Figure 9:**
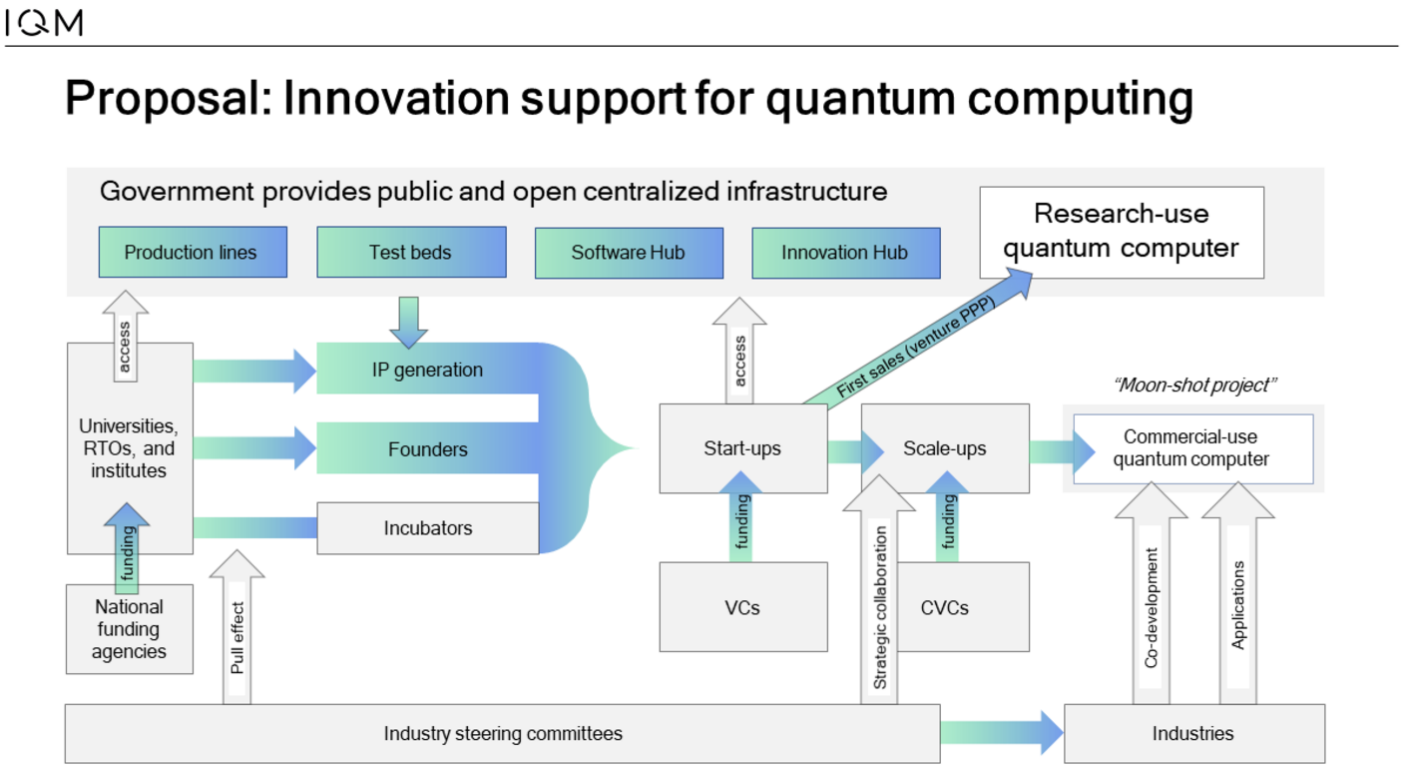
Our proposal for generic innovation support on a state level. Acronyms used are: research and technology organization (RTO), venture-capital firms (VCs), and corporate venture capital firms (CVCs). The picture shows how different partners come together to form a sample ecosystem. There are different roles at different stages of the process for partners

Figure 9 below shows our proposal for the way to implement innovation support mechanisms for a quantum-computing ecosystem. Such innovation support mechanisms are important because they support the creation and existence of ecosystems.

Contrary to many other proposals and models, we propose that the ecosystems for quantum computing are built around leading startups because the EU is missing large technology companies that can serve as the system integrator in such cases. The startup focus ensures that the commercial utilization of the technologies is at the heart of the effort to create and keep such an ecosystem. This is important since only by relying on a commercial approach it is possible to ensure that the ecosystems stay vibrant and successfully answer to the needs of the EU.

We argue that likely the most beneficial strategy is to put certain leading unicorn startup companies at the center of such efforts. These companies have the highest likelihood of providing a major global impact. In our view, a developed EU-based quantum-computing ecosystem is of high international standing, resilient, innovative, inclusive and can attract talent into the EU. All these parts are important, but perhaps the most important one is the high standing. For the public capital invested we need to aim for world-class results, otherwise the funds may be wasted in a commercial sense. Companies that are not internationally successful in their field cannot produce the economic results required to fund additional investments into the field as well as any future such fields.

### Support collaborations between developed industries and startups

As stated in Sect. 4.4, the cooperation between industries and startups is very important. Thus, measures to increase such actions needs to be supported. Namely, EU-based industries require incentivization to partner with EU-based quantum-computing startup companies in a manner that does not violate the relevant regulations for state aid. For example, the creation of joint development agreements between startups and established companies may be subsidized to help the startups with their revenue creation.

This point is strongly tied to the ability of EU-based quantum-computing startups to create commercial traction. Large industrial companies do generally not engage in cooperation with startups without a clear business case or a very clear promise of one in the short term. The teams aiming to become the future quantum unicorns need to learn how to communicate their value in other ways than what is required in the academia.

Cooperation between startups and companies generally takes the form of proof-of-concept projects, where a business problem is addressed with a solution. In quantum computing, such a case is built by understanding the capabilities of the solution offered by the startup as well as understanding the problem present at the established industrial company. Overall, training on new developments and emerging fields such as quantum computing is an example of an area of cooperation that can be had between established and startup companies. For the ability of Europe to scale quantum computing as an industry, and deep technology in general, we need to train a whole new generation of business-minded talent for the field.

### Provide equity-style funding to scale companies

**Figure 10:**
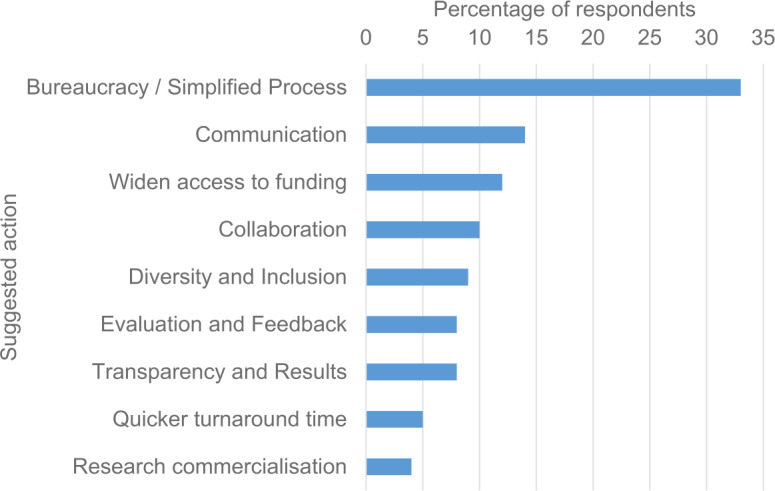
Relative number of answers to a question: “What would you recommend the European Commission do to improve the efficiency and effectiveness of its research and innovation programs?” The question was asked from 670 respondents by Atomico and some respondents provided multiple recommendations for the question. The data are obtained from Reference [28]

The amount of bureaucracy tied to EU-sponsored funding is one of the main concerns among startup founders as shown in Fig. 10 below.

The EU already has a great way to grant equity-style funding to startups in the form of the European Innovation Council (EIC) accelerator. We argue that such funding instruments should continue and be further enlarged in terms of their budget. From a free-market point of view, these efforts do not conflict with private capital, but leverage it. The reason is that in many cases the capital needs for quantum-computing companies are so high that they cannot be provided by only private investors. Having specialized deep-technology funds sponsored by the EU focusing on quantum can help to convince venture capitalists to invest in quantum because they can share the risks with a large fund and provide flexibility in the investment size and terms. In addition, the EU may consider establishing a pool of certified quantum experts who help startups in the due-diligence process of their investment rounds.

Naturally, one way to improve such efforts is to simplify the overall process of applying for such programs. The desire for such changes seems clear from the data shown in Fig. 10.

Furthermore, we stress the importance of a long-term commitment from the EU in the field of quantum computing. In the current environment of low interest rates, more capital is flowing to alternative investments such as the venture capital investments into deep-technology companies [29]. This development may not last for long. Thus, it is very important that if private money decreases as a funder of risky deep-technology ventures, the EU and the member states step up their role.

### Increase the efforts for intellectual property standardization

The EU should be encouraging and leading the efforts for increased standardization in quantum computing. In technologies such as those in the third and fourth-generation cellular-phone networks European companies led the world in terms of standardization. Such a scenario is desirable also in the case of quantum computing. However, to reach this ambitious target, strong support for EU-based standards is required from the EU.

We propose that the EU engages further with expert groups to explore how it can foster the standardization of quantum-computing solutions in the EU. We see that such efforts can, not only lead to EU-based quantum unicorns, but also to significant technology synergies when the EU has its standards for quantum computers.

The creation of new businesses and the strengthening of promising SMEs can be supported by an enhanced technology transfer between research organization and industry. One platform to coordinate such actions is the Quantum Industry Consortium. This consortium is well-suited to build a platform for standardization and IP protection.

An idea to be explored here is the possibility of launching a program for a pool of intellectual property where participating companies can license their quantum-computing solutions. This may take the form of one or multiple patent pools where participants from the community license intellectual property belonging to them which is required to implement certain quantum technologies. For example, companies may choose to license their IP regarding a specific subsystem of a quantum computer. Such a pool may in consequence even create a European standard platform for quantum computing.

The benefit of a patent or IP pool for EU-based quantum-computing companies lie in the enabled and enhanced abilities to negotiate international licensing deals and to defend against patent litigations as well as in the shared impact that the standardization brings to the development efforts of multiple companies working in the field. If EU-based companies work together, they can also expect more success in terms of international adoption of their specific inventions compared with the case where they work alone.

Naturally, any efforts regarding the patent pool should be tied to standardization. Thus, when a standard is reached, the IP pool will place emphasis on standard essential IP. We stress that such an arrangement, if implemented correctly, can produce increased cooperation between the EU-based quantum-computing companies and research organizations since the IP pool renders the IP situation for specific areas clear.

## Conclusions and outlook

In this paper, we discussed the current state of support for quantum-computing industry and startups in the EU as well as the existing measures used or proposed to this end. In addition, we have proposed new measures, which in our understanding, will likely result in increased number of quantum-computing unicorns in the EU. The measures we proposed are for the EU and the member states to:

1. Create world-class quantum hubs in Europe.
2. Build manufacturing infrastructure such as clean rooms.
3. Act as an active purchaser of quantum computing solutions.
4. Build ecosystems around startups.
5. Support collaboration between startups and established companies.
6. Provide equity-style funding for scaling quantum computing companies.
7. Support IP standardization efforts

In conclusion, Europe and the EU have all the required potential for the creation of world-leading quantum-computing unicorn companies. This potential may realize into societal prosperity especially provided that the EU will consider new measures to support quantum-computer companies, concentrate funding in both academia and in the commercial efforts to the most successful ventures, and set quantum computing as one of the major focus areas of its future technology strategy.

## Data Availability

The data presented in this paper are available via the links presented in the references list, except for the data for Fig. 1, which can be provided by the authors upon request.

## Abbreviations

EU: European Union
SME: Small and medium-sized enterprise
IP: Intellectual property
COVID-19: Coronavirus disease 2019
R&D: Research and development
GDP: Gross domestic product
RTO: Research and technology organization
VCs: Venture capital firms
CVCs: Corporate venture capital firms
EIC: European Innovation Council

## References

[1] World Economic Forum. Bridging the gap in European scale-up funding: the green imperative in an unprecedented time. http://www3.weforum.org/docs/WEF_Bridging_the_Gap_in_European_Scale_up_Funding_2020.pdf. Accessed: 07.12.2020.

[2] A unicorn company is a privately owned startup or growth company that has a valuation in excess of 1 billion USD.

[3] McKinsey Q. A game plan for quantum computing. https://www.mckinsey.com/business-functions/mckinsey-digital/our-insights/a-game-plan-for-quantum-computing. Accessed: 17.12.2020.

[4] Inside Quantum Technology. Quantum computing market to reach US$1.9 billion by 2023, says new IQT report. https://www.insidequantumtechnology.com/news-release/quantum-computing-market-to-reach-us1-9-billion- by-2023-says-new-iqt-report/#:~:text=Quantum%20Computing%20Market%20To%20Reach,2023%2C%20Says%20 New%20IQT%20Report. Accessed: 08.12.2020.

[5] BCG. Where will quantum computers create value – and when? https://www.bcg.com/publications/2019/quantum-computers-create-value-when. Accessed: 02.12.2020.

[6] Kiltz A. The European quantum computing startup landscape. https://medium.com/uvc-partners-news/ the-european-quantum-computing-startup-landscape-a115ffe84ad8#:~:text=The%2069%20European%20 quantum%20computing,over%20%E2%82%AC150m%20to%20date. Accessed: 02.12.2020.

[7] Tracxn. Quantum computing startups in United States. https://tracxn.com/explore/Quantum-Computing-Startups-in-United-States. Accessed: 07.12.2020.

[8] Quantum Technologies Flagship High-level Steering Committee. Quantum technologies flagship final report. http://ec.europa.eu/newsroom/document.cfm?doc_id=46979. Accessed: 17.12.2020. P. 3.

[9] Qureca. Overview on quantum initiatives worldwide. https://www.qureca.com/overview-on-quantum-initiatives-worldwide/. Accessed: 03.02.2021.

[10] The figure is based on data that has been collected from the FamPat database of Questel-Orbit on Wednesday 2 December 2020 by listing patent applications that were filed on 1 January 2001 or later and classified by appropriate patent authorities into at least one of the CPC patent classes G06N-010/00 (“Quantum computers, i.e. computer systems based on quantum-mechanical phenomena”). Note that the data for 2019 might still be incomplete.

[11] Zhou H. China: patent subsidy In China: a reformation from quantity to quality. https://www.mondaq.com/china/patent/779124/patent-subsidy-in-china-a-reformation-from-quantity-to-quality. Accessed: 08.12.2020.

[12] Figure based on data provided by Atomico in: Atomico, The State of European Tech 2019. https://2019.stateofeuropeantech.com/chapter/investments/article/investment-industry/. Accessed: 02.12.2020. P. 30.

[13] Figure based on data provided by Atomico in: Atomico, The State of European Tech 2019. https://2019.stateofeuropeantech.com/chapter/investments/article/investment-industry/. Accessed: 02.12.2020. P. 31.

[14] Mind the bridge, tech scaleup Europe 2019 report. https://startupeuropepartnership.eu/wp-content/uploads/2019/06/2019_TechScaleupEurope.pdf. Accessed: 17.12.2020. P. 6.

[15] World Economic Forum. Bridging the gap in European scale-up funding: the green imperative in an unprecedented time. http://www3.weforum.org/docs/WEF_Bridging_the_Gap_in_European_Scale_up_Funding_2020.pdf. Accessed: 07.12.2020.

[16] For the role of U.S. government driving commercialization see e.g.: Wessner C. Public/private partnerships for innovation: experiences and Perspective from the U.S. http://www.oecd.org/sti/inno/2730122.pdf. Accessed: 02.12.2020.

[17] Figure based on data provided by Atomico in: Atomico, The State of European Tech 2019. https://2019.stateofeuropeantech.com/chapter/investments/article/investment-industry/. Accessed: 02.12.2020. P. 241.

[18] Figure based on data provided by Atomico in: Atomico, State of European Tech 2020. https://2020.stateofeuropeantech.com/chapter/regulation-policy/article/policy-and-regulation/. Accessed: 08.12.2020.

[19] Figure based on data provided by Atomico in: Atomico, State of European Tech 2020. https://2020.stateofeuropeantech.com/chapter/regulation-policy/article/policy-and-regulation/. Accessed: 08.12.2020.

[20] Defense Advanced Research Projects Agency. https://www.darpa.mil/. Accessed: 02.12.2020.

[21] European Commission. Proposal for a council regulation on establishing the European high performance computing joint undertaking. https://ec.europa.eu/digital-single-market/en/news/proposal-council-regulation-establishing-european-high-performance-computing-joint-0. Accessed: 02.12.2020.

[22] European Commission. EU’s next long-term budget & NextGenerationEU: key facts and figures. https://ec.europa.eu/info/sites/info/files/about_the_european_commission/eu_budget/mff_factsheet_agreement_en_web_20.11.pdf. Accessed: 02.12.2020. P. 3.

[23] Figure based on the Eurostat, R&D expenditure data. https://ec.europa.eu/eurostat/statistics-explained/index.php?title=File:Gross_domestic_expenditure_on_R%26D,_2008_-2018_(%25,_relative_to_GDP)_final_F1.png. Accessed: 02.12.2020. Note that (1) data for Japan contains breaks in the series for years 2008, 2013 and 2018; (2) data for United States excludes most or all capital expenditure, the definition differs in years 2008-2016 and 2018; and data are provisional for year 2017; (3) in the data for China there is a break in the series in the year 2009.

[24] Cavalleri M, Eliet A, McAdam P, Petroulakis F, Soares A, Vansteenkiste I. Concentration, market power and dynamism in the euro area. https://www.ecb.europa.eu/pub/pdf/scpwps/ecb.wp2253~cf7b9d7539.en.pdf. Accessed: 02.12.2020. P. 24.

[25] For Nokia’s role in the Finnish R&D spending relative to GDP see: ALI-YRKKÖ, Jyrki, & HERMANS, Raine. Nokia in the Finnish Innovation System. Helsinki, ETLA, Elinkeinoelämän Tutkimuslaitos, The Research Institute of the Finnish Economy, 19.6.2002, 35 p. (Keskusteluaiheita, Discussion Papers; ISSN 0781-6847; no. 811). https://www.etla.fi/wp-content/uploads/2012/09/dp811.pdf. Accessed: 02.12.2020. For the dwindling of R&D spending relative to GDP see: Official Statistics of Finland (OSF): Research and development [e-publication]. ISSN=2342-6721. 2017, 1. Research and development 2017. Helsinki: Statistics Finland [referred: 2.12.2020]. http://www.stat.fi/til/tkke/2017/tkke_2017_2018-10-25_kat_001_en.html.

[26] Figure based on data provided by Atomico in: Atomico, State of European Tech 2020. https://2020.stateofeuropeantech.com/chapter/investments/article/investments-geo-industry/. Accessed: 08.12.2020.

[27] European Commission. Quantum technologies flagship. https://ec.europa.eu/digital-single-market/en/quantum-technologies-flagship. Accessed: 02.12.2020.

[28] Figure based on data provided by Atomico in: Atomico, State of European Tech 2020. https://2020.stateofeuropeantech.com/chapter/regulation-policy/article/policy-and-regulation/. Accessed: 08.12.2020.

[29] For the overall deep-technology boom see: Dealroom.co, 2021: the year of deep tech. https://blog.dealroom.co/2021-the-year-of-deep-tech/. Accessed: 17.02.2021.

